# Pioneering terahertz blood analysis: Hollow-core PCF with optimized sensitivity and low loss

**DOI:** 10.1371/journal.pone.0319860

**Published:** 2025-03-25

**Authors:** A.H.M. Iftekharul Ferdous, Md Shahadat Hosen, Kayab Khandakar, Diponkar Kundu, Most. Momtahina Bani, Khalid Sifulla Noor, Suchana Aktar Tithi

**Affiliations:** Department of Electrical and Electronic Engineering, Pabna University of Science and Technology, Pabna,Bangladesh; DIT University, INDIA

## Abstract

Blood detection is crucial for the human body. Its detection is very crucial and sensitive. In this paper, a hollow core photonic crystal fiber (PCF) biosensor operating in the terahertz frequency range is proposed. The building blocks of this proposed biosensor’s hexagonal cladding structure are the same square-shaped air gaps in the cladding and core. Hemoglobin, white blood cells (WBC), red blood cells (RBC), plasma and water are among the analytes that fill the core. The sensing aspects of the design will be examined using the finite element method. The COMSOL v6.1a software simulation findings show that the sensitivity for water is 93.08 percent, for plasma it is 94.55%, for hemoglobin it is 96.21%, for WBC it is 95.16%, and for RBC it is 97.05 percent. The suggested design’s detection has the lowest confinement loss at frequencies between f =  1 and 2.8 THz. In addition to these, the design exhibits, under ideal design circumstances, very low and flattened dispersion, huge beam divergence, improved effective area, substantial birefringence, and negligible effective material loss. This proposed PCF biosensor is a viable option for employment in various practical applications due to its simple shape and great detecting capacity. PCF offer significant benefits for blood component analysis due to their unique structure and light-guiding properties. By enabling precise control over light-matter interaction, PCFs can be highly sensitive to the presence and characteristics of different blood components, such as red and white blood cells, platelets, hemoglobin, and glucose. This has major implications in medical diagnostics, offering advantages in speed, sensitivity, and minimally invasive testing.

## 1. Introduction

The method used to determine the quantity of cholesterol present in human body. Therefore, there is pressing need for effective cholesterol identification method. Numerous methods have been used to study biological substances, including analytes, enzymes, antibodies, nucleic acids, and related chemicals. Biosensors based on PCF are becoming more and more common these days. They are employed in the identification of many components of blood, including water, hemoglobin, white blood cells (WBC) and red blood cells (RBC) [1]. The over 4,000 components that make up human blood are extremely useful [2]. The blood circulates throughout the body, providing fuel and oxygen to the cells as well as aiding. A biosensor is a device that looks for the existence of additional chemicals or biological substances in the disposal of waste. Because blood contains so many different types of cells and proteins, it is thicker than water. The blood content of an average human body is around 5L or 55% of this blood is made up of a liquid known as plasma. Water makes up 99 percent of this plasma, with the remaining material consisting of the following substances [3]. The squishy and pulpy fat in our bodies is called cholesterol. Cholesterol maintains the fluidity of cell membranes and helps to maintain the human body’s structural soundness. In addition, it controls the production of vitamin D. The human body mostly obtains cholesterol from eggs, milk, meat, and other sources. Nonetheless, having too much cholesterol in our bodies raises the chance of developing heart disease, high blood pressure, and stroke. As a result, starting at age 35 for males and age 45 for women, we need to get frequent checkups to determine the amount of cholesterol in our bodies. There are benefits and drawbacks to cholesterol for health. Due to its presence in our bodies, low-density lipoprotein (LDL) cholesterol may result in heart attacks and strokes. Hence, LDL cholesterol is unhealthy for humans. Once more, our bodies use high-density lipoprotein (HDL) cholesterol to help eliminate low-density lipoprotein (LDL) cholesterol. HDL cholesterol is hence beneficial to human health. In human blood, total cholesterol levels are typically under 200 milligrams per deciliter, whereas LDL and HDL concentrations are typically 70–130 mg and 40–60 mg respectively. A higher amount of HDL cholesterol is preferable to a lower amount of LDL cholesterol. As of right now, the most common method for determining biochemical characteristics is a blood test, such as chromatographic [4], voltametric [5], or electrometric [6]. These perceptions technologies do have certain disadvantages though, such as the need for skilled labor (operation assistants), lengthy inquiry times, and sophisticated measurement procedures. These factors explain why scientists are still working to develop an effective method of sensing biochemicals. Researchers have known for the past 20 years that the PCF may produce effective results, particularly for sensing applications. Lately, many kinds of PCF sensors have been studied in the liquid, gas, and areas of chemical sensing [7–11]. Researchers, engineers, academics, businesspeople, and others have been interested in chemical sensing research, particularly in THz domain. THz sensing was introduced by Jepsen et al [12] early in 2017 where chemical sensing is carried out. The THz frequency band is the range of electromagnetic waves between 0.1 and 10 THz, which falls in between microwaves and infrared. Many diverse and improved applications, including sensing [13,14], security [15], communications [16], pharmaceutical drug testing [17], biological sensing [18], imaging [19], and environmental applications [20], are increasingly gaining attention as potential candidates for the THz signal. Surface Plasmon Resonance (SPR) sensor is a highly sensitive optical device that combines the guiding properties of PCF with SPR phenomena to detect changes in refractive index. It is widely used for chemical, biological, and environmental sensing applications due to its enhanced sensitivity and compact design. The interaction between the plasmonic material and the analyte ensures precise detection of small variations in target substances [21–23].

Recently, A PCF with a heptagonal shape was invented by Hasan *et al* [20]. They proposed that the highest sensitivity for benzene is approximately 63.04% when it comes to perceiving water, ethanol, and benzene. Kamani *et al.* conduct the identical experiment [24] for an alternative PCF structure, where they suggested a benzene sensitivity of about 77.08%. Ahmed together with others [24] introduced a blood component detecting sensor, and for red blood cells, they proposed a sensitivity of roughly 80.93%. Islam along with others [25] introduced a blood component detecting sensor, and for red blood cells, they proposed a sensitivity of roughly 80.93%. Islam along with others [26] have revealed that they were able to identify the harmful substances having sensitivity of 94.4% using rectangular hollow-core PCF sensor. Thus, there is still potential to improve chemical detection performance inside the THz range utilizing PCF [27–29].

PCF sensors provide distinct advantages for analyzing blood components in real-time applications because of their distinct optical characteristics and high sensitivity. In medical field, particularly in diagnostics and point-of-care (POC) testing, PCF sensors are emerging as valuable tools for detecting and monitoring various blood parameters identify different blood components. This proposed sensor managed to acquire sensitivity values of 93.08%, 94.55%, 96.21%, 95.16%, and 97.05% for water, plasma, hemoglobin, WBC, and red blood cells. This sensor also measured confinement loss and found that the same analytes produced results of 1.92 × 10^ − 01^ dB/m, 1.54 ×  10^ − 02^ dB/m, 5.3 ×  10^ − 04^ dB/m, 6.3 ×  10^ − 03^ dB/m, and 3.8 ×  10^ − 05^ dB/m. Some more optical parameters are added in this research paper such as EML, total loss, numerical aperture (NA), Spot Size, Birefringence.

The novelty of the proposed PCF structure lies in its unique combination of a hybrid core with Zeonex cladding and rectangular air holes, which enhances light confinement and interaction with the analyte. Unlike traditional PCF designs that often rely on circular or hexagonal air holes, this structure’s distinct geometry offers improved sensitivity and versatility for sensing applications. Additionally, the integration of a Perfectly Matched Layer (PML) minimizes simulation losses, optimizing performance. This approach distinguishes the sensor from other reported PCF sensors by providing more efficient light guidance and better detection capabilities for a wide range of chemicals and biological samples.

## 2. Methodology

**Fig 1:**
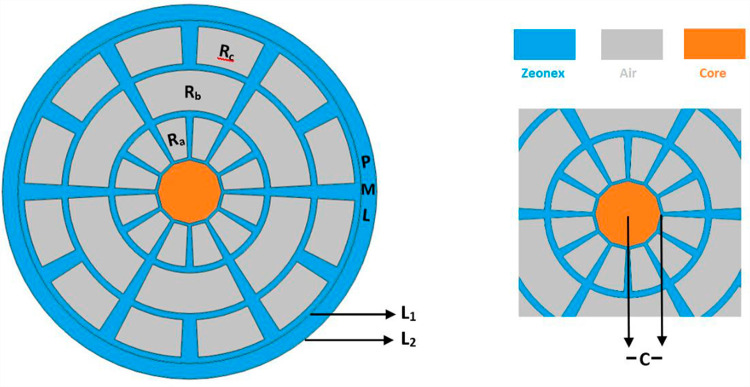
The suggested PCF structure, with the core depicted in an enlarged view.

COMSOL Multiphysics Version 6.1a was used to model this PCF geometry. Finite element method (FEM) is utilized in the context of Multiphysics to study a variety of optical characteristics of PCF. When compared to other analytical approaches, FEM is the most comprehensive numerical methodology because of its practical advantages, which include quicker processing, component optimization, and a reduction in the physical prototype. The geometrical configuration is seen in Fig 1 with the core enlarged. contrasting a hollow core with a de-decanal structure with a square, rectangular, pentagonal, hexagonal, etc. While maintaining the same cladding structure, very little performance difference was found when comparing the optical properties of the proposed PCF to those of a circular-core and a de-decanal-core PCF in order to boost the relative sensitivity in the center region. The hollow core PCF was selected because it can enable increased sensitivity due to its larger interaction area between applied analytes and light intensity. However, it aids in minimizing the contact between light and material, which lowers EML. In order to use the refractive index to determine the various components of the core, blood is introduced to the appropriate location within the hollow area. In order to compare our findings with previous comparable studies in the PCF context, we optimized the design parameters and adjusted the geometrical parameters in almost all previous works.

While the core hole radius is C =  P, the optimal PCF overall diameter is fixed at 12P. There are six air holes in the second layer and twelve in the first and third layers of the three layers of cladding. R_a1_ =  1.1P is the inner radius of the first layer, while R_a2_ =  2.4P is the outer layer. R_b1_ =  2.6P is the inner radius of the second layer, while R_b2_ =  3.9P is the outer layer. R_c1_ =  4.1P is the inner radius of the third layer, while R_c2_ =  5.3P is the outer radius. Two layers of air holes are separated by 0.2P. The P magnitude in this instance falls between 120 and 210 μm.

To avoid ecological contamination, a layer known as a perfectly matched layer (PML) is applied to the outermost area. PML functions as a cover that is resistant to reflection by restraining the waves that are leaving the fiber [30]. Here, the thickness of PML is L = 0.5P, where the inner radius L_1_ = 5.5 and the outer layer is L_2_ = 6. Zeonex is allowed to serve as this model’s backdrop material because it has minimal material absorption loss and dispersion, moisture insensitivity in a constant RI =  1.53 [31,32]. Furthermore, zeonex has a higher crystal transition temperature and more chemical restiveness, which aid in the flexible fabrication of the fiber. Zeonex is preferred over topas, silica, and other fiber materials for the reasons mentioned above.

The proposed PCF structure is designed with a hybrid core and Zeonex cladding, featuring rectangular air holes for enhanced light guidance and analyte interaction. To design this structure, simulation tools like COMSOL Multiphysics can be used to optimize parameters such as the air hole radius (Ra, Rb), pitch, and core geometry to achieve desired optical properties. The inclusion of a Perfectly Matched Layer (PML) ensures minimal loss during simulations. For experimentation, fabrication techniques like stack-and-draw or 3D printing can be employed to realize the structure, followed by coating the inner cladding with a plasmonic material like gold or silver for SPR-based sensing. The sensor can then be tested by introducing analytes of varying refractive indices to analyze its sensitivity and accuracy. This design enables high sensitivity for chemical and biological sensing applications, providing a foundation for practical implementation and performance evaluation.

**Fig 2:**
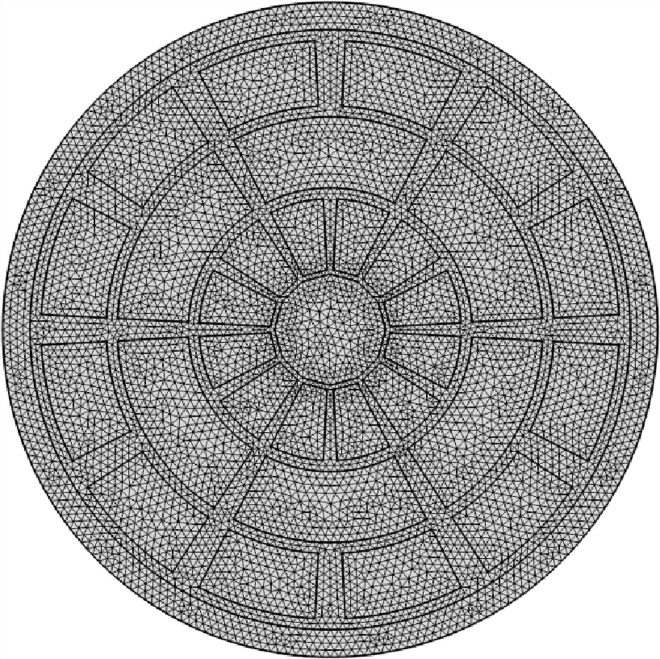
Mesh of the proposed design.

**Fig 3:**
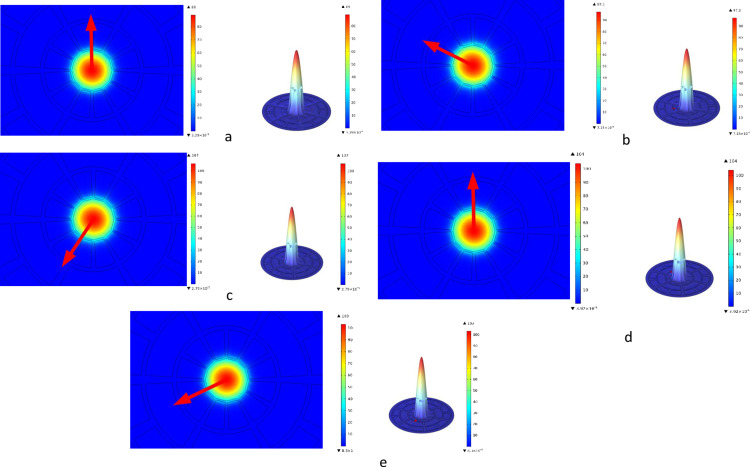
The suggested PCF’s power distribution of modes for several targeted chemicals at a frequency of 2 THz, along with the intensity scale: (a) Water (b) Plasma (c) WBC (d) Hemoglobin (e) RBC.

An organized configuration of air holes called a mesh affects the fiber wrapping’s spectroscopic properties within a PCF device. This mesh’s tiny size and flexible shape improve responsiveness for applications such as gas detection, notably adaptive vision, by enabling customizable illumination steering. In addition to 140 vertex elements and 1696 boundary units, Mesh provides a total of 11972 components. 0.5728 is the lowest feature grade. Fig 2 shows the mesh arrangement based on the additional PCF. First, Fig 3 shows the relationship between blood substances, ideal design parameters (see Sect. 2), and light intensity at a working frequency of 2 THz. It is clear that the light intensity has had a significant effect on the blood components and that the center region is densely illuminated. On the other hand, some touch is seen in the cladding region.

In the sensor of PCF, the electric field distribution is crucial to its sensing capabilities. The unique structure of PCFs, with air holes surrounding a solid or hollow core, enables them to confine and manipulate light in distinct ways This has an immediate effect on the dispersion of the electric field,The interaction between the electric field and the analyte in sensor is a key factor in sensing applications, such as refractive index sensing, gas detection, and biosensing. In the sensor of PCF, density distribution typically pertains to how the density of materials within the fiber structure is organized across the cross-sectional area, particularly in the arrangement of the air holes and solid or hollow core [33]. The distribution of density in PCFs is designed to achieve specific light-guiding properties and to enhance interactions with external analytes, making it highly relevant in sensing applications. The spatial density distribution within the PCF plays a critical role in how it confines and guides light, as well as in the sensor’s overall sensitivity.

The power and density distribution figure for a photonic crystal fiber (PCF) sensor is crucial for understanding its performance and efficiency. It illustrates how electromagnetic energy propagates through the core and cladding regions, highlighting the sensor’s capability to confine light effectively. This distribution directly affects the sensitivity of the sensor, as higher power confinement in the core enhances interaction with the analyte. A well-distributed power density minimizes confinement loss and ensures robust signal detection. Additionally, the figure provides insights into mode field characteristics, such as spot size and numerical aperture, which are vital for optimizing sensing accuracy. It aids in identifying potential design improvements, such as adjusting air-hole geometry or pitch, to enhance the PCF’s interaction with specific analytes. Overall, this figure is indispensable for evaluating and optimizing PCF sensor designs for precise and efficient sensing applications.

## 3. Results and analyses

The PCF’s cross-section shape is discretized into smaller attainable components using FEM. These components can analyze the features associated with light transmission in the solution of the equations proposed by Maxwell. By using suitable boundary conditions and material parameters, FEM offers accurate computational solutions for intricate PCF architectures. It can explain several characteristics, including irregular solids and air gaps. This technique is essential to comprehending light containment and dispersal features, forecasting oscillatory properties, and improving PCF construction.

The term “RS” in PCF describes how sensitive the fiber is to variations in its surroundings, including humidity, stress, and the outside medium’s RI. It measures the effect that changes in these variables have regarding the directed modes’ dispersion properties inside the PCF. For detecting uses, RS is essential since a higher RS denotes more advanced detecting capacities. Usually, a variation in the actual RI or other mechanical qualities as a result of outside factors is compared to compute it. To improve the functioning of PCF-based detectors, RS must be optimized.

Use the following mathematical equations to determine RS [34].


$$r=\frac{n_{r}}{n_{eff}}\times p\%$$ (1)

Here, p represent the percentage of power overlap.


$$p=\frac{\int_{sample}R_{e}\left(E_{x}H_{y}-E_{y}H_{x}\right)dxdy}{\int_{total}R_{e}\left(E_{x}H_{y}-E_{y}H_{x}\right)dxdy}$$ (2)

On the other hand, E_x_ and H_x_ represents the x component of electric and magnetic field respectively.

The relative sensitivity of your sensor varies with changes in frequency and pitch due to the interaction between the fiber structure and the terahertz wave contact with the blood sample. Changes in the confinement of the guided mode and the overlap of the evanescent field with the sample cause sensitivity to vary with frequency. By changing the photonic bandgap and the fiber’s ability to confine light, pitch variations have an impact on the mode field distribution and how it interacts with the sample. Higher frequencies typically lead to greater confinement, which reduces sensitivity, whereas optimized pitch values improve field overlap with the sample, increasing sensitivity. This paired dependency results in a complex sensitivity fluctuation over pitch and frequency. Relative sensitivity (RS) represents the efficiency with which the guided light in a photonic crystal fiber (PCF) sensor interacts with the analyte. It indicates the proportion of light energy that is effectively confined to the sensing region, directly influencing the sensor’s ability to detect changes in the analyte’s properties. If RS is high, it means a significant portion of the light is engaged in sensing, allowing the detection of subtle variations in the analyte, such as refractive index changes caused by the presence of specific chemicals or biological markers. For instance, when RS is 0.94, it reflects strong light-analyte interaction, enhancing the precision and reliability of the detection process. A high RS ensures better sensitivity, enabling the sensor to identify even minute differences in analyte composition. This makes it an essential factor in optimizing PCF sensors for applications in chemical, biological, and environmental sensing, where accurate and efficient detection is critical.

**Fig 4:**
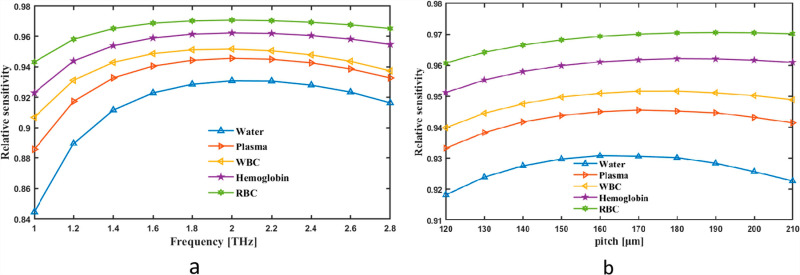
Provide demonstrations of how the RS affects (a) frequency in THz and (b) pitch in µm.

The RS was first conceived as a simultaneous frequency and pitch indicator. RS frequency fluctuation changes between 1 and 2.8 THz. The sensing ratings related to the y-pole was 93.04%, 94.55%,95.16%,96.21 and 97.05 for Water, Plasma, WBC, Hemoglobin and RBC in 2 THz frequency respectively are seen in Fig 4(a). The range of values in RS may be changed by Pitch, as shown in Fig 4(b). Fig 4 demonstrates the variation of relative sensitivity (RS) with frequency (in THz) and pitch (in µm) for different analytes. Subplot (a) shows that RS increases with frequency before stabilizing, indicating stronger light-analyte interaction at higher frequencies. Subplot (b) illustrates how RS slightly improves with increasing pitch, though the effect becomes less significant beyond a certain point. RBCs consistently exhibit higher RS compared to water, indicating a stronger interaction with the guided light. Practically, this means the sensor is more sensitive and precise in detecting RBCs, making it highly effective for blood analysis or disease detection. Such insights help in optimizing sensor parameters like frequency and pitch to achieve maximum sensitivity for specific analytes, enabling the development of highly accurate and application-specific sensors.

The measurement of light retardation resulting from inherent material absorption as well as scattering inside PCF is known as EML. It takes into consideration how the core and cladding materials—as well as the periodic air-hole structure—combine to affect the total loss. Understanding and reducing losses during transmission in PCFs is crucial for industries like high-power lasers and telecommunications that demand low-loss laser transmission. This is made possible by EML. To find the EML, which provides details on the fiber’s performance and efficacy, the imaginary part of the complicated RI may be integrated throughout the fiber’s cross-sectional area.

Use the following mathematical equations to determine EML [35].


$$\alpha_{eff}=\frac{{\left(\frac{\varepsilon_{0}}{\mu_{0}}\right)}^{\frac{1}{2}}\int_{A_{max}}n\alpha_{mat}{\left|E\right|}^{2}dA}{2\int_{ALL}S_{z}dA}$$ (3)

E stands for the modulated electric field, and αmat for a dominant context element’s loss coefficient. The decrease in the element’s power is caused by this loss coefficient.

**Fig 5:**
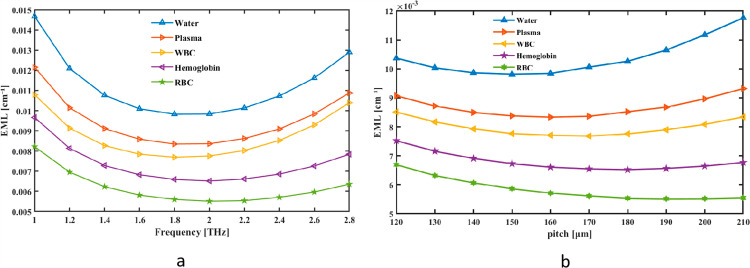
Provide demonstrations of how EML affects (a) frequency in THz and (b) pitch in µm.

The interaction of multiple elements causes your terahertz blood analysis sensor’s Effective Mode Loss (EML) to vary with frequency. Firstly, the loss is greatly affected by the frequency-dependent absorbed terahertz radiation by blood proteins and water. In the hollow-core PCF, dispersion results in changes in confinement efficiency and mode propagation across frequencies. Third, when more energy leaks into the cladding because of decreased core-mode localization, confinement loss rises at higher frequencies. Fourth, as frequency variations, the mode attenuation is modified by the interaction between the blood sample and the evanescent field. Finally, the fiber’s structural flaws and scattering losses intensify changes in loss that are depending on frequency. We first thought of the EML as a simultaneous pitch and frequency indicator. Changes inside EML are shown as frequency fluctuation between 1 and 2.8 THz. The tool measures the fiber’s rate at 2 THz EML, which is 0.0098383 cm^ − 1^ for Water, 0.0083631 cm^−1^ for Plasma, 0.0077545 cm^−1^ for WBC, 0.0065164 cm^−1^ for Hemoglobin and 0.0055109 cm^−1^ for RBC in Fig 5(a). Fig 5(b), on the other hand, shows how pitch may alter the range of values in EML. EML graph for pitch and frequency illustrates how material damping or energy dissipation varies across frequencies in relation to pitch. It typically shows that material losses are frequency-dependent, with higher losses at specific frequency ranges due to increased vibrational energy absorption. This graph can help in understanding the impact of material properties on sound attenuation, particularly in acoustic design and engineering applications.

CL refers to the loss of electromagnetic radiation due to leakage through the cladding or surrounding materials. This leakage occurs when photons cannot be effectively contained inside the fiber’s core due to arrangement of its refractive crystal structure. Variables like the RI difference between cladding and core, the PCF’s design of the wavelength light influence CL. Minimizing CL is crucial for optimizing PCF performance across various applications, including optical sensing and telecommunications. To reduce CL and improve fiber efficiency, strategies such as careful design of the PCF structure and refinement of the manufacturing process are employed.

Use the following mathematical equations to determine CL [36].


$$L_{c}=\frac{40\pi }{\ln\left(10\right)\lambda }img\left(n_{eff}\right)\times \frac{10^{6}dB}{m}$$ (4)

Furthermore, the real wavelength is shown by λ, while the area of electromagnetic interference is represented by imaging n_eff_.

**Fig 6:**
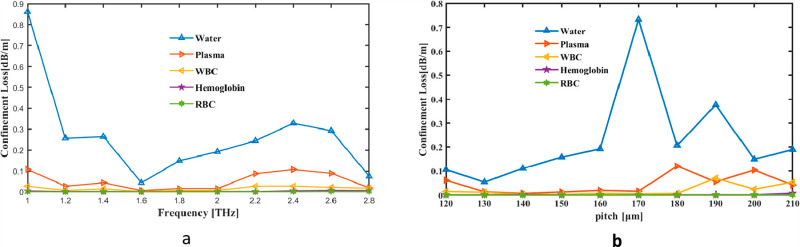
Provide demonstrations of how the CL affects (a) frequency in THz and (b) pitch in µm.

The CL was first thought of as a simultaneous pitch and frequency indicator. Changes in CL are shown as a frequency variation between 1 and 2.8 THz. The tool measures the fiber’s rate at 2 THz CL, which is 1.921 × 10^−01^ dBm^−1^ for Water, 1.5471 × 10^−02^ dBm^−1^ for Plasma, 6.3463 × 10^−03^ dBm^−1^ for WBC, 5.3585 × 10^−04^ dBm^−1^ for Hemoglobin and 3.8599 × 10^−05^ dBm^−1^ for RBC in Fig 6(a). Instead Fig 6(b) illustrates, Pitch may change the range of values in CL. CL graph for pitch and frequency depicts the energy loss due to imperfect confinement of acoustic waves in a system, such as waveguides or resonators. It typically shows higher confinement losses at certain frequency ranges where the wave energy leaks or interacts with boundaries inefficiently. The trends in the graph can provide insights into the design and optimization of structures to minimize energy loss and improve acoustic performance.

The variation of confinement loss with frequency and pitch in your sensor is influenced by how the guided mode interacts with the hollow-core PCF structure. As frequency increases, the mode becomes less confined due to higher energy leakage into the cladding, leading to increased confinement loss. The pitch affects the photonic bandgap properties and the periodicity of the cladding, altering the fiber’s ability to trap light within the core. Smaller pitches typically enhance confinement by creating stronger reflection at the core-cladding boundary, reducing loss, while larger pitches may weaken confinement, increasing loss. This interplay between frequency-dependent mode behavior and structural design determines the confinement loss variation.

The numerical aperture (NA) of a fiber is a crucial metric that shows how well it can absorb and restrict light. It shows the sine of the light cone’s maximum half-angle that may enter or leave the fiber. The refractive index (RI) difference between the core and cladding and the structural properties of the photonic crystal lattice both affect the NA. Higher NA values in PCFs enable better light confinement, enhancing their performance for applications such as high-quality image processing, sensing, and telecommunications. Optimizing NA is essential for tailoring the light-guiding properties of PCFs to specific applications.

Use the following mathematical equations to determine NA [37].


$$NA=\frac{1}{\sqrt{1+\frac{\pi A_{eff}f^{2}}{c^{2}}}}\approx \frac{1}{\sqrt{1+\frac{\pi A_{eff}}{\lambda^{2}}}}$$ (5)

A_eff_ represents EA and PCF, while λ denotes the wavelengths that are of interest or utility.Top of FormBottom of Form

**Fig 7.**
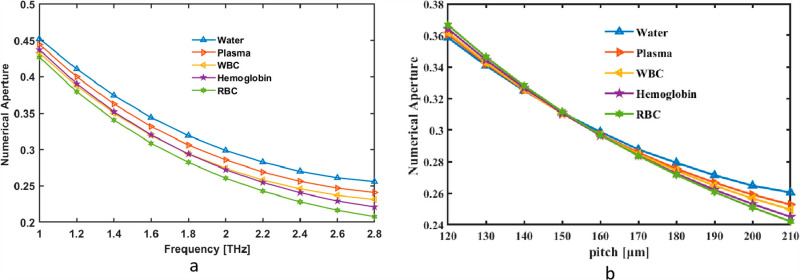
Provide demonstrations of how NA affects (a) frequency in THz and (b) pitch in µm.

Initially, we thought of the NA as a simultaneous pitch and frequency indicator. Changes inside NA are shown as frequency fluctuation between 1 and 2.8 THz. The tool measures the fiber’s rate at 2 THz EML, which is 0.298 for Water, 0.285 for Plasma, 0.274 for WBC, 0.272 for Hemoglobin and 0.260 for RBC in Fig 7(a). In contrast, Fig 7(b) illustrates how pitch may change the range of values in NA. NA graph for pitch and frequency illustrates the capability of a system, such as an optical or acoustic waveguide, to collect or confine energy at various frequencies. It typically shows that NA varies with frequency, often increasing for higher frequencies where the system can focus or confine waves more effectively. This relationship helps in understanding the directional and focusing efficiency of waveguides or resonators in different frequency ranges.

A hollow-core PCF numerical aperture (NA) fluctuates with pitch and frequency as a result of modifications to the fiber’s light confinement and propagation properties. The core-guided modes interact with the photonic crystal cladding in different ways as the frequency rises, changing the effective mode area and, in turn, the NA. Likewise, changes in pitch (the separation between air holes in the cladding) alter the photonic bandgap structure and affect how well the fiber confines light. Larger pitches may decrease the NA, whilst lower pitches tend to increase mode confinement. For terahertz blood analysis applications, this interaction guarantees low loss and sensitivity optimization.

The longitudinal section of a fiber where light energy is efficiently contained in the core is referred to as the EA. It quantifies the spatial distribution of the photonic type supported by the fiber. Factors like the RI contrast, geometry, and the length of the PCF’s photonic crystal structure significantly influence EA. Typically, EA in PCFs is larger than in conventional fibers, which offers advantages such as reduced ripple effects and improved power handling. Enhancing EA is crucial for applications requiring optimal photon confinement, such as high-power laser delivery and advanced optical fields like communication systems, to achieve peak efficiency.

Use the following mathematical equations to determine EA [38].


$$A_{eff}=\frac{{\left[\int I\left(r\right)rdr\right]}^{2}}{{\left[\int I^{2}\left(r\right)rdr\right]}^{2}}$$ (6)

Thus, when I(r) =  | E | 2, the electric field is represented across the detection element.

**Fig 8:**
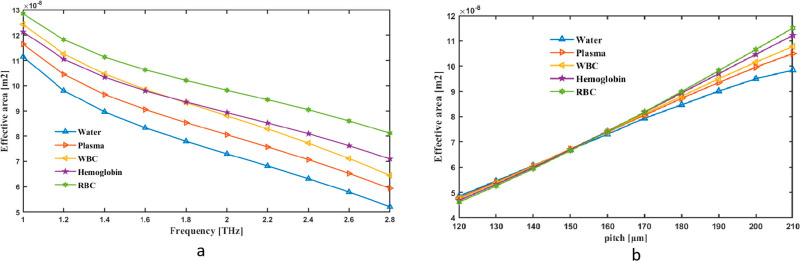
Provide demonstrations of how EA affects (a) frequency in THz and (b) pitch in µm.

We first thought of the EA as pitch and frequency indicator that worked together. EA changes are shown as frequency variation between 1 and 2.8 THz. The tool measures the fiber’s rate at 2 THz CL, which is 7.30 × 10^−08^ m^2^ for Water, 8.05 × 10^−08^ m^2^ for Plasma 8.80 × 10^−08^ m^2^ for WBC, 8.93 × 10^−08^ m^2^ for Hemoglobin and 9.82 × 10^−08^ m^2^ for RBC in Fig 8(a). Conversely, Fig 8(b) shows how Pitch may alter the range of values in EA. EA graph for pitch and frequency demonstrates how the distribution of energy or influence varies across frequencies relative to pitch perception. It often shows that certain frequency ranges, such as mid to high frequencies, have a broader effective area, indicating a stronger contribution to pitch perception. This reflects how the auditory system processes frequencies with varying sensitivities, aligning with the critical band theory and cochlear mechanics.

Due to their effects on mode confinement and field distribution, frequency and pitch cause a hollow-core photonic crystal fiber’s (PCF) effective area to vary. Higher frequencies cause the light’s wavelength to drop, strengthening its interaction with the fiber structure. As the mode gets more closely confined, this can lower the effective area. However, changes in pitch, which controls the distance between air holes in the cladding, change the photonic bandgap characteristics of the fiber. Generally speaking, a higher pitch weakens mode confinement and increases the effective area, while a lower pitch increases mode confinement and decreases the effective area. In terahertz blood analysis applications, these fluctuations are essential for maximizing the sensor’s sensitivity and reducing losses.

The spot size refers to the measurement of the diameter of the smallest focused beams or spots created when sunlight passes through a PCF. The PCF’s optical crystal’s structural characteristics, core dimensions, and refractive index contrast all affect this size. Smaller spot sizes in PCFs enable precise photon confinement, which is advantageous for applications like high-quality imaging, monitoring, and microscopy. Adjusting the spot size in PCFs allows for control over the spectral properties of the transmitted light, which is essential for optimizing performance across various optical devices.

Use the following mathematical equations to determine Spot Size [39].


$$W_{eff}=R\times \left(.65\times 1.619\times V^{-1.5}+2.789\times V^{-6}\right)$$ (7)

These symbols respectively denote the central radius (R) and the cutoff frequency (V) of a component.

**Fig 9:**
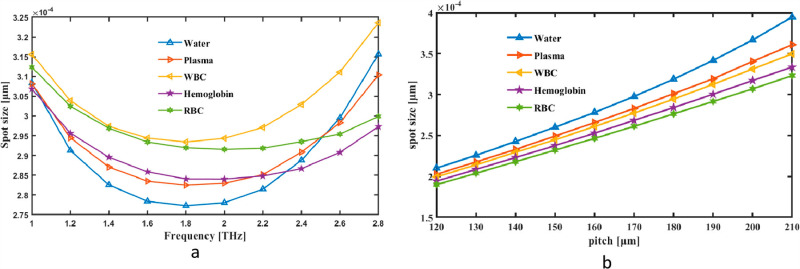
Provide demonstrations of how Spot Size affects (a) frequency in THz and (b) pitch in µm.

The Spot Size was first thought of as a simultaneous pitch and frequency indicator. Spot changes are shown as frequency variation between 1 and 2.8 THz. The tool measures the fiber’s rate at 2 THz CL, which is 2.78 × 10^−04^ μm for Water, 2.82 × 10^−04^ μm for Plasma 2.94 × 10^−04^ μm for WBC, 2.84 × 10^−04^ μm for Hemoglobin and 2.91 × 10^−04^ μm for RBC in Fig 9(a). Fig 9(b), on the other hand, shows how Pitch may alter the range of values in Spot. A spot size graph for pitch and frequency represents the spatial extent or beam diameter of acoustic or optical waves as a function of frequency. Typically, the graph shows that spot size decreases with increasing frequency, as higher frequencies tend to focus energy into smaller regions due to shorter wavelengths. This trend is crucial in applications requiring precise energy confinement, such as acoustic imaging or waveguide design.

Because frequency and pitch affect mode confinement and beam divergence, they alter the spot size of a hollow-core PCF. At higher frequencies, the spot size decreases due to tighter mode confinement caused by the shorter wavelength. On the other hand, lower frequencies enable the mode to propagate, hence expanding the spot size. The photonic bandgap and confinement qualities are affected by changes in pitch, which determine the distance between air holes in the cladding. A bigger pitch results in a larger spot size because it weakens confinement, whereas a smaller pitch increases confinement and reduces the spot size. In applications involving terahertz blood analysis, these modifications are essential for maximizing sensitivity and reducing losses.

In PCFs, all factors that contribute to the decrease in light intensity during fiber propagation are included in the Total Loss (TL). These factors include substrate absorption, scattering losses, confinement losses, and bending losses. Dispersion losses arise from imperfections or defects in the fiber’s molecular structure, while material absorption is due to the fiber’s inherent absorption characteristics. Bending losses occur when the fiber is curved, leading to photon leakage, and confinement losses result from inadequate light confinement within the core. To ensure effective light transmission and optimize fiber performance for applications such as high-power laser delivery, monitoring, and telecommunications, it is crucial to minimize total loss in PCFs. This is achieved through various optimization techniques, including material selection, design considerations, and manufacturing processes.

Use the following mathematical equations to determine TL [36].Top of FormBottom of Form


$$TotalLoss=\alpha_{eff}+L_{c}$$ (8)

Here, $\alpha_{eff}$ and $L_{c}$ be the EML and CL respectively.

**Fig. 10.**
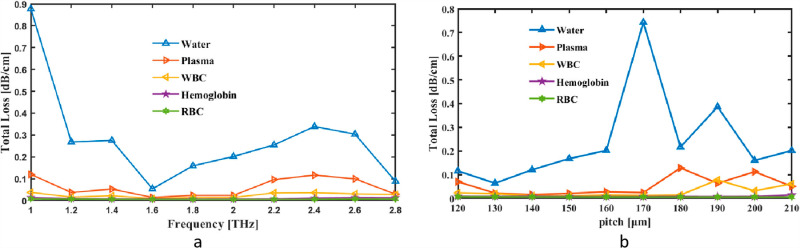
Provide demonstrations of how the Total Loss affects (a) frequency in THz and (b) pitch in µm.

Initially, TL was seen as a simultaneous pitch and frequency indicator. Modifications inside TL are shown as frequency fluctuation between 1 and 2.8 THz. The tool measures the fiber’s rate at 2 THz CL, which is 2.02 × 10^−01^ dB/cm for Water, 2.38 × 10^−02^ dB/cm for Plasma 1.41 × 10^−02^ dB/cm for WBC, 7.05 × 10^−03^ dB/cm for Hemoglobin and 5.54 × 10^−03^ dB/cm for RBC in Fig 10(a). Nevertheless, Fig 10(b) illustrates how Pitch may change the TL value range. TL graph for pitch and frequency illustrates the cumulative energy losses in a system as a function of frequency, encompassing factors like material loss, confinement loss, and scattering. The graph typically shows that total loss increases at certain frequency ranges, reflecting heightened dissipation or inefficiencies in energy propagation. Such trends are essential for optimizing system design, minimizing losses, and improving performance in acoustic or waveguide applications

The total loss in a hollow-core PCF varies with frequency and pitch due to their effect on light confinement and scattering. At higher frequencies, the shorter wavelength interacts more strongly with the fiber structure, potentially increasing scattering losses if the design is not optimized. Similarly, variations in pitch, which determines the spacing between the cladding air holes, influence the photonic bandgap’s ability to confine light within the core. A smaller pitch enhances confinement, reducing leakage losses, while a larger pitch may allow more light to escape into the cladding, increasing total loss. Optimizing these parameters is essential for achieving low loss in terahertz blood analysis applications.

The term “Birefringence (Bi)” refers to the phenomenon in PCFs where electromagnetic pulses travel at different speeds along orthogonal axes due to changes in the index of refraction (RI) of the fiber core with respect to polarization direction. This results in the radiation splitting into two orthogonal polarization components: a fast axis and a slow axis. Birefringence in PCFs is typically caused by physical asymmetries or stresses within the fiber, such as geometric imperfections or external pressure. High birefringence PCFs are essential for applications that are sensitive to polarization, such as polarization-sensitive sensors and fiber-optic gyroscopes. Careful design and manufacturing techniques enable the control and optimization of birefringence in PCFs, allowing for tailored polarization properties suited to specific applications.

Use the following mathematical equations to determine Birefringence [35].


$$Birefringence=\left|n_{x}-n_{y}\right|$$ (9)

Accordingly, the total difference between the effective RI of the x and y polarized phases defines birefringence.

**Fig 11.**
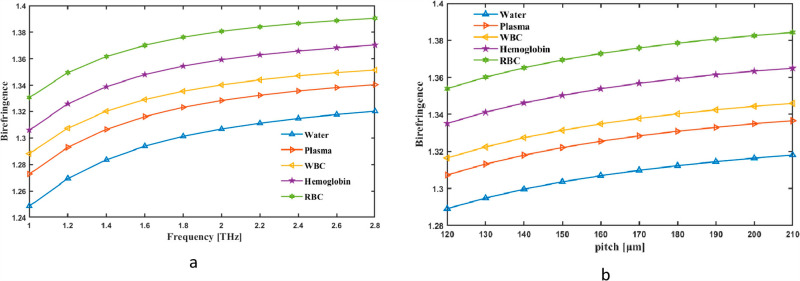
Provide demonstrations of how the Bi affects (a) frFequency in THz and (b) pitch in µm.

We first thought of the Bi as a simultaneous pitch and frequency indicator. Changes inside Bi are shown as frequency fluctuation between 1 and 2.8 THz. The tool measures the fiber’s rate at 2 THz CL, which is 1.3069 for Water, 1.3284 for Plasma 1.3403 for WBC, 1.3593 for Hemoglobin and 1.3807 for RBC in Fig 11(a). Conversely Fig 11(b) shows how pitch may alter the range of values in Bi. The Bi graph for pitch and frequency illustrates the variation in refractive indices for two orthogonal polarization modes as a function of frequency. The graph typically shows that birefringence increases or varies nonlinearly with frequency due to the dispersion properties of the material or system. This behavior is critical for understanding polarization effects, wave propagation characteristics, and optimizing birefringent materials for applications like acoustic filters or optical waveguides.

Because frequency and pitch affect mode asymmetry and structural anisotropy, they alter the birefringence of a hollow-core PCF. If asymmetry is present, the shorter wavelength at higher frequencies increases the guided modes’ sensitivity to the fiber’s geometric features, resulting in increased birefringence. The distribution of the refractive index contrast is also influenced by changes in pitch, which establish the distance between air holes in the cladding. While a higher pitch tends to decrease birefringence by making the mode field more uniform, a smaller pitch can increase it by enhancing the asymmetry in mode confinement. In terahertz blood analysis applications, this tunability is essential for maximizing polarization control. Table 1 summarizes the primary attributes of the current and of the PCF sensors that we have investigated.

**Table 1:** Comparison with the earlier suggested sensor that was included into the PCF technology to evaluate the components of blood.

Ref.	Relative Sensitivity					Lc	NA	EML
	RBCs	Hemoglobin	WBCs	Plasma	Water			
[**40**]	56.05	66.47	53.72	54.05	55.09	10^−10 1± ^ dB/m	–	–
[**41**]	66.46	65.05	62.72	58.04	55.82	10^−14.5 ± 1.5^ dB/m	–	–
[**3**]	80.03	80.56	80.13	79.91	79.39	10^−13.5 ± 0.5^ dB/m	–	–
[**42**]	93.50	92.41	91.25	90.48	89.14	10^−13.5 ± 0.5^ dB/m	–	–
[**25**]	95.80	95	93.6	92.5	91.4	10^−10 1 ±^ dB/m	0.38	–
**Proposed work**	97.05	96.21	95.16	94.55	93.08	10^−13.5 ± 0.5^ dB/m	0.248 to 0.298	0.0055 to 0.0098

PCF fabrication is a process that involves creating specialized optical fibers with periodic structures along the fiber’s cross-section. PCFs are designed with unique properties that allow them to guide light differently from conventional fibers, and they have applications in various fields, including telecommunications, sensing, and medical imaging. Key Steps in PCF Fabrication are 1) Designing the Fiber Structure. 2) Preform Fabrication 3) Drawing the Fiber 4) Post-Processing. There are two types of PCFs Fabrication one is Solid Core PCFs and the other is Hollow Core PCFs. Fabricating PCFs requires advanced precision and control over material quality and hole structure, as variations can significantly affect fiber performance. The fabrication of PCF sensor involves a stack-and-draw method. First, silica capillaries are arranged in a preform to create the desired cladding structure, incorporating air holes for light guidance. This preform is heated and drawn into a fine fiber while maintaining the structural geometry. For specific sensing applications, materials like Zeonex or porous structures can be integrated to enhance sensitivity. Advanced techniques such as sol-gel processes or doping may be applied for functionalization. Precision in the fabrication process is critical to achieving the designed geometrical parameters, ensuring optimal optical performance and high detection accuracy [9,43].

## 4. Conclusion

In order to distinguish between distinct blood components based on the RI in the THz spectrum, this research presents a PCF design. The suggested PCF obtains maximum sensitivity values of 97.05% for RBCs, 96.21% for hemoglobin, 95.16% for white blood cells (WBCs), 94.55% for plasma, and 93.08% for water at the selected operating frequency of 2 THz and under ideal geometric circumstances. Furthermore, the PCF exhibits very low confinement losses at the same frequency: 3.8 ×  10^ – 05^ dB/m for red blood cells, 5.35 ×  10 – ^04^ dB/m for hemoglobin, 6.34 ×  10^ – 03^ dB/m for white blood cells, 1.54 ×  10^ – 02^ dB/m for plasma, and 1.92 ×  10^ – 01^ dB/m for water. Furthermore, the suggested PCF is ideal for advanced sensing applications because to its excellent numerical aperture, near-zero flattening dispersion throughout a broad THz range, and low material absorption loss. Its practical potential has also been shown by an evaluation of the viability of fabricating it using existing technical techniques. All things considered, this study offers a PCF design that exhibits exceptional optical performance in the THz range and enhanced capacity to identify different blood components in comparison to earlier designs.

## Supporting Information

S1 File Frequency.
(PDF)

S2 File Pitch.
(PDF)
