# Supplementary material for: Pioneering terahertz blood analysis: Hollow-core PCF with optimized sensitivity and low loss
Source: PLoS One. 2025 Mar 25;20(3):e0319860. doi: 10.1371/journal.pone.0319860 (PMC11936239; doi:10.1371/journal.pone.0319860)
Supplement: S1 File — (PDF) [file pone.0319860.s001.pdf]

wave	freq	p	nr	l	aff	real-x pol
#DIV/0!						
3.00E-04	1.00E+12	1.60E-04	1.33	0.0002451	0.95	1.24860000000000
2.50E-04	1.20E+12	1.60E-04	1.33	0.0002451	0.95	1.26930000000000
2.14E-04	1.40E+12	1.60E-04	1.33	0.0002451	0.95	1.28360000000000
1.88E-04	1.60E+12	1.60E-04	1.33	0.0002451	0.95	1.29380000000000
1.67E-04	1.80E+12	1.60E-04	1.33	0.0002451	0.95	1.30130000000000
1.50E-04	2.00E+12	1.60E-04	1.33	0.0002451	0.95	1.30690000000000
1.36E-04	2.20E+12	1.60E-04	1.33	0.0002451	0.95	1.31130000000000
1.25E-04	2.40E+12	1.60E-04	1.33	0.0002451	0.95	1.31490000000000
1.15E-04	2.60E+12	1.60E-04	1.33	0.0002451	0.95	1.31780000000000
1.07E-04	2.80E+12	1.60E-04	1.33	0.0002451	0.95	1.32020000000000
#DIV/0!						
#DIV/0!						
#DIV/0!						
3.00E-04	1.00E+12	1.70E-04	1.35	0.000260419	0.95	1.27280000000000
2.50E-04	1.20E+12	1.70E-04	1.35	0.000260419	0.95	1.29300000000000
2.14E-04	1.40E+12	1.70E-04	1.35	0.000260419	0.95	1.30660000000000
1.88E-04	1.60E+12	1.70E-04	1.35	0.000260419	0.95	1.31620000000000
1.67E-04	1.80E+12	1.70E-04	1.35	0.000260419	0.95	1.32320000000000
1.50E-04	2.00E+12	1.70E-04	1.35	0.000260419	0.95	1.32840000000000
1.36E-04	2.20E+12	1.70E-04	1.35	0.000260419	0.95	1.33240000000000
1.25E-04	2.40E+12	1.70E-04	1.35	0.000260419	0.95	1.33570000000000
1.15E-04	2.60E+12	1.70E-04	1.35	0.000260419	0.95	1.33830000000000
1.07E-04	2.80E+12	1.70E-04	1.35	0.000260419	0.95	1.34050000000000
#DIV/0!						
#DIV/0!						
3.00E-04	1.00E+12	1.80E-04	1.36	0.000275738	0.95	1.28830000000000
2.50E-04	1.20E+12	1.80E-04	1.36	0.000275738	0.95	1.30750000000000
2.14E-04	1.40E+12	1.80E-04	1.36	0.000275738	0.95	1.32020000000000
1.88E-04	1.60E+12	1.80E-04	1.36	0.000275738	0.95	1.32910000000000
1.67E-04	1.80E+12	1.80E-04	1.36	0.000275738	0.95	1.33550000000000
1.50E-04	2.00E+12	1.80E-04	1.36	0.000275738	0.95	1.34030000000000
1.36E-04	2.20E+12	1.80E-04	1.36	0.000275738	0.95	1.34400000000000
1.25E-04	2.40E+12	1.80E-04	1.36	0.000275738	0.95	1.34700000000000
1.15E-04	2.60E+12	1.80E-04	1.36	0.000275738	0.95	1.34940000000000
1.07E-04	2.80E+12	1.80E-04	1.36	0.000275738	0.95	1.35140000000000
3.00E-04	1.00E+12	1.80E-04	1.38	0.000285896	0.985	1.30610000000000
2.50E-04	1.20E+12	1.80E-04	1.38	0.000285896	0.985	1.32580000000000
2.14E-04	1.40E+12	1.80E-04	1.38	0.000285896	0.985	1.33890000000000
1.88E-04	1.60E+12	1.80E-04	1.38	0.000285896	0.985	1.34790000000000
1.67E-04	1.80E+12	1.80E-04	1.38	0.000285896	0.985	1.35440000000000
1.50E-04	2.00E+12	1.80E-04	1.38	0.000285896	0.985	1.35930000000000
1.36E-04	2.20E+12	1.80E-04	1.38	0.000285896	0.985	1.36300000000000
1.25E-04	2.40E+12	1.80E-04	1.38	0.000285896	0.985	1.36590000000000
1.15E-04	2.60E+12	1.80E-04	1.38	0.000285896	0.985	1.36830000000000

1.07E-04	2.80E+12	1.80E-04	1.38	0.000285896	0.985	1.37030000000000
3.00E-04	1.00E+12	1.90E-04	1.4	0.000301779	0.985	1.33070000000000
2.50E-04	1.20E+12	1.90E-04	1.4	0.000301779	0.985	1.34940000000000
2.14E-04	1.40E+12	1.90E-04	1.4	0.000301779	0.985	1.36170000000000
1.88E-04	1.60E+12	1.90E-04	1.4	0.000301779	0.985	1.37010000000000
1.67E-04	1.80E+12	1.90E-04	1.4	0.000301779	0.985	1.37620000000000
1.50E-04	2.00E+12	1.90E-04	1.4	0.000301779	0.985	1.38070000000000
1.36E-04	2.20E+12	1.90E-04	1.4	0.000301779	0.985	1.38410000000000
1.25E-04	2.40E+12	1.90E-04	1.4	0.000301779	0.985	1.38680000000000
1.15E-04	2.60E+12	1.90E-04	1.4	0.000301779	0.985	1.38890000000000
1.07E-04	2.80E+12	1.90E-04	1.4	0.000301779	0.985	1.39070000000000
#DIV/0!						

imag-x pol	area-x pol	power-x pol	sens-x pol
			#DIV/0!
4.7409000000000E-06	1.1149000000000E-07	7.9292000000000E-01	0.8446128463880
1.1737000000000E-06	9.8009000000000E-08	8.4907000000000E-01	0.8896739147562
1.0377000000000E-06	8.9629000000000E-08	8.7983000000000E-01	0.9116343876597
1.5054000000000E-07	8.3292000000000E-08	8.9780000000000E-01	0.9229200803834
4.5532000000000E-07	7.7981000000000E-08	9.0853000000000E-01	0.9285675094137
5.2816000000000E-07	7.3018000000000E-08	9.1468000000000E-01	0.9308473486877
6.1086000000000E-07	6.8193000000000E-08	9.1757000000000E-01	0.9306551513765
7.5189000000000E-07	6.3205000000000E-08	9.1748000000000E-01	0.9280161228991
6.1792000000000E-07	5.7839000000000E-08	9.1496000000000E-01	0.9234305660950
1.4694000000000E-07	5.2109000000000E-08	9.0960000000000E-01	0.9163520678685
			#DIV/0!
			#DIV/0!
			#DIV/0!
5.9244000000000E-07	1.1643000000000E-07	8.3502000000000E-01	0.8856670333124
1.2354000000000E-07	1.0445000000000E-07	8.7865000000000E-01	0.9173839907193
1.7173000000000E-07	9.6501000000000E-08	9.0274000000000E-01	0.9327253941528
2.0335000000000E-08	9.0508000000000E-08	9.1691000000000E-01	0.9404562376539
4.7827000000000E-08	8.5391000000000E-08	9.2548000000000E-01	0.9442246070133
4.2523000000000E-08	8.0562000000000E-08	9.3043000000000E-01	0.9455589430894
2.1852000000000E-07	7.5781000000000E-08	9.3259000000000E-01	0.9449088111678
2.4599000000000E-07	7.0709000000000E-08	9.3259000000000E-01	0.9425743056075
1.8912000000000E-07	6.5275000000000E-08	9.3045000000000E-01	0.9385843981170
3.6532000000000E-08	5.9352000000000E-08	9.2613000000000E-01	0.9326933979858
			#DIV/0!
			#DIV/0!
1.4921000000000E-07	1.2428000000000E-07	8.5906000000000E-01	0.9068707599162
3.1326000000000E-08	1.1256000000000E-07	8.9524000000000E-01	0.9311865391969
5.4425000000000E-08	1.0462000000000E-07	9.1528000000000E-01	0.9428728980458
6.0615000000000E-09	9.8501000000000E-08	9.2709000000000E-01	0.9486437438868
1.8855000000000E-08	9.3217000000000E-08	9.3401000000000E-01	0.9511445900412
1.7443000000000E-08	8.8095000000000E-08	9.3787000000000E-01	0.9516550026114
6.8520000000000E-08	8.2874000000000E-08	9.3931000000000E-01	0.9504922619048
6.3415000000000E-08	7.7265000000000E-08	9.3873000000000E-01	0.9477897550111
4.5454000000000E-08	7.1145000000000E-08	9.3622000000000E-01	0.9435743293316
3.4777000000000E-08	6.4515000000000E-08	9.3140000000000E-01	0.9373272162202
2.3185000000000E-08	1.2130000000000E-07	8.7347000000000E-01	0.9228915090728
3.7132000000000E-09	1.1051000000000E-07	9.0674000000000E-01	0.9438084175592
1.6277000000000E-09	1.0332000000000E-07	9.2541000000000E-01	0.9538171633430
2.3787000000000E-09	9.7977000000000E-08	9.3656000000000E-01	0.9588640106833
5.6791000000000E-10	9.3502000000000E-08	9.4348000000000E-01	0.9613130537507
1.4728000000000E-09	8.9351000000000E-08	9.4772000000000E-01	0.9621522842640
1.6920000000000E-09	8.5249000000000E-08	9.4996000000000E-01	0.9618083639032
1.0970000000000E-08	8.0949000000000E-08	9.5061000000000E-01	0.9604230177905
1.3512000000000E-08	7.6245000000000E-08	9.4999000000000E-01	0.9581131330849

1.1227000000000E-08	7.1007000000000E-08	9.4797000000000E-01	0.9546804349413
1.7286000000000E-09	1.2843000000000E-07	8.9638000000000E-01	0.9430615465544
2.5463000000000E-10	1.1819000000000E-07	9.2339000000000E-01	0.9580154142582
6.0238000000000E-11	1.1134000000000E-07	9.3865000000000E-01	0.9650510391422
2.1281000000000E-10	1.0625000000000E-07	9.4788000000000E-01	0.9685657981169
4.1060000000000E-11	1.0206000000000E-07	9.5360000000000E-01	0.9700915564598
1.0609000000000E-10	9.8202000000000E-08	9.5720000000000E-01	0.9705801405084
5.7712000000000E-11	9.4423000000000E-08	9.5922000000000E-01	0.9702391445705
9.7431000000000E-10	9.0442000000000E-08	9.6001000000000E-01	0.9691476781079
3.2152000000000E-09	8.6064000000000E-08	9.5980000000000E-01	0.9674706602347
3.2292000000000E-09	8.1140000000000E-08	9.5863000000000E-01	0.9650406270224
			#DIV/0!

EML-x pol	confinement -x pol	NA- x pol	spot-x pol
	#DIV/0!	#DIV/0!	#DIV/0!
0.0146750000000	8.624515975728E-01	4.52135000388969E-01	3.083920449499E-04
0.0120950000000	2.562195633921E-01	4.10772673699166E-01	2.912630927708E-04
0.0107660000000	2.642857752582E-01	3.74446405780490E-01	2.825438380765E-04
0.0100930000000	4.381732194262E-02	3.44151773310208E-01	2.784092986237E-04
0.0098281000000	1.490950305917E-01	3.19121533362203E-01	2.772982978492E-04
0.0098383000000	1.921628533713E-01	2.98870402850140E-01	2.780456874782E-04
0.0101330000000	2.444771672816E-01	2.82604417476567E-01	2.814843844522E-04
0.0107310000000	3.282762673918E-01	2.70091297981704E-01	2.887911350707E-04
0.0116210000000	2.922668347883E-01	2.61280891610804E-01	2.995231414811E-04
0.0128980000000	7.484658729198E-02	2.55984980300998E-01	3.156628773111E-04
	#DIV/0!	#DIV/0!	#DIV/0!
	#DIV/0!	#DIV/0!	#DIV/0!
	#DIV/0!	#DIV/0!	#DIV/0!
0.0121490000000	1.077750689671E-01	4.44370577541245E-01	3.081794850916E-04
0.0101450000000	2.696887182539E-02	3.99992258784730E-01	2.944588867115E-04
0.0091128000000	4.373691450814E-02	3.62682883343694E-01	2.870653133613E-04
0.0085880000000	5.918860380651E-03	3.31717539361365E-01	2.835097691530E-04
0.0083482000000	1.566100331220E-02	3.06317620467455E-01	2.824783548989E-04
0.0083631000000	1.547133636381E-02	2.85730572587856E-01	2.829893498560E-04
0.0086219000000	8.745563728902E-02	2.69161027093822E-01	2.851454967135E-04
0.0090924000000	1.073995917165E-01	2.56351937150107E-01	2.908603773462E-04
0.0098405000000	8.945090593468E-02	2.46910713926585E-01	2.982871516916E-04
0.0108790000000	1.860824504526E-02	2.40822256486293E-01	3.103935868099E-04
	#DIV/0!	#DIV/0!	#DIV/0!
	#DIV/0!	#DIV/0!	#DIV/0!
0.0107920000000	2.714387624161E-02	4.32815253329741E-01	3.155864005088E-04
0.0091345000000	6.838488576996E-03	3.87553352980344E-01	3.038656335948E-04
0.0082683000000	1.386118658421E-02	3.50117360532215E-01	2.973764558499E-04
0.0078441000000	1.764306476386E-03	3.19403175701787E-01	2.944040896129E-04
0.0076903000000	6.174090314080E-03	2.94339006784248E-01	2.934137268182E-04
0.0077545000000	6.346365971213E-03	2.74200009825249E-01	2.943521707151E-04
0.0080264000000	2.742293733774E-02	2.58186680803083E-01	2.971092711234E-04
0.0085264000000	2.768708121753E-02	2.45921651426520E-01	3.029201620819E-04
0.0092931000000	2.149905604037E-02	2.37102539010312E-01	3.111249554812E-04
0.0103930000000	1.771430356781E-02	2.31522993798262E-01	3.236212615391E-04
0.0096650000000	4.217751964759E-03	4.37094889220976E-01	3.068276128304E-04
0.0081340000000	8.105942598512E-04	3.90587709045476E-01	2.956418589736E-04
0.0072838000000	4.145494424090E-04	3.52041727348359E-01	2.895569499408E-04
0.0068260000000	6.923625860563E-04	3.20168819415157E-01	2.858583838435E-04
0.0065938000000	1.859627488873E-04	2.93928894008380E-01	2.840462648209E-04
0.0065164000000	5.358555181106E-04	2.72409975353479E-01	2.840381534384E-04
0.0066146000000	6.771688554504E-04	2.54801501869999E-01	2.848020280935E-04
0.0068576000000	4.789517952477E-03	2.40591851017194E-01	2.866713378361E-04
0.0072614000000	6.390972086450E-03	2.29467300435667E-01	2.907945083425E-04

0.0078384000000	5.718678613904E-03	2.21228312349813E-01	2.973115667043E-04
0.0082162000000	3.144621973812E-04	4.27059487082605E-01	3.123539936060E-04
0.0069545000000	5.558591414034E-05	3.79570436766768E-01	3.024493250637E-04
0.0062336000000	1.534166573191E-05	3.40649642694933E-01	2.968976171352E-04
0.0058156000000	6.194210364428E-05	3.08685890462779E-01	2.934023115326E-04
0.0056036000000	1.344514178182E-05	2.82360389174685E-01	2.919474403937E-04
0.0055109000000	3.859920689595E-05	2.60717258879560E-01	2.915325832840E-04
0.0055409000000	2.309738119725E-05	2.42874441312523E-01	2.918245461206E-04
0.0057076000000	4.253851628330E-04	2.28309993043044E-01	2.935094092312E-04
0.0059678000000	1.520741078475E-03	2.16632770611759E-01	2.954277195178E-04
0.0063571000000	1.644852318520E-03	2.07589348875341E-01	2.998911545302E-04
	#DIV/0!	#DIV/0!	#DIV/0!
