# Supplementary material for: Pioneering terahertz blood analysis: Hollow-core PCF with optimized sensitivity and low loss
Source: PLoS One. 2025 Mar 25;20(3):e0319860. doi: 10.1371/journal.pone.0319860 (PMC11936239; doi:10.1371/journal.pone.0319860)
Supplement: S2 File — (PDF) [file pone.0319860.s002.pdf]

wave	freq	p	nr	l	aff	real-x pol
#DIV/0!						
1.50E-04	2.00E+12	1.20E-04	1.33	0.000183825	0.95	1.28910000000000
1.50E-04	2.00E+12	1.30E-04	1.33	0.000199144	0.95	1.29480000000000
1.50E-04	2.00E+12	1.40E-04	1.33	0.000214463	0.95	1.29960000000000
1.50E-04	2.00E+12	1.50E-04	1.33	0.000229781	0.95	1.30360000000000
1.50E-04	2.00E+12	1.60E-04	1.33	0.0002451	0.95	1.30690000000000
1.50E-04	2.00E+12	1.70E-04	1.33	0.000260419	0.95	1.30980000000000
1.50E-04	2.00E+12	1.80E-04	1.33	0.000275738	0.95	1.31230000000000
1.50E-04	2.00E+12	1.90E-04	1.33	0.000291056	0.95	1.31450000000000
1.50E-04	2.00E+12	2.00E-04	1.33	0.000306375	0.95	1.31640000000000
1.50E-04	2.00E+12	2.10E-04	1.33	0.000321694	0.95	1.31810000000000
#DIV/0!						
#DIV/0!						
#DIV/0!						
1.50E-04	2.00E+12	1.20E-04	1.35	0.000183825	0.95	1.30730000000000
1.50E-04	2.00E+12	1.30E-04	1.35	0.000199144	0.95	1.31320000000000
1.50E-04	2.00E+12	1.40E-04	1.35	0.000214463	0.95	1.31800000000000
1.50E-04	2.00E+12	1.50E-04	1.35	0.000229781	0.95	1.32210000000000
1.50E-04	2.00E+12	1.60E-04	1.35	0.0002451	0.95	1.32550000000000
1.50E-04	2.00E+12	1.70E-04	1.35	0.000260419	0.95	1.32840000000000
1.50E-04	2.00E+12	1.80E-04	1.35	0.000275738	0.95	1.33090000000000
1.50E-04	2.00E+12	1.90E-04	1.35	0.000291056	0.95	1.33300000000000
1.50E-04	2.00E+12	2.00E-04	1.35	0.000306375	0.95	1.33500000000000
1.50E-04	2.00E+12	2.10E-04	1.35	0.000321694	0.95	1.33660000000000
#DIV/0!						
#DIV/0!						
1.50E-04	2.00E+12	1.20E-04	1.36	0.000183825	0.95	1.31650000000000
1.50E-04	2.00E+12	1.30E-04	1.36	0.000199144	0.95	1.32240000000000
1.50E-04	2.00E+12	1.40E-04	1.36	0.000214463	0.95	1.32740000000000
1.50E-04	2.00E+12	1.50E-04	1.36	0.000229781	0.95	1.33140000000000
1.50E-04	2.00E+12	1.60E-04	1.36	0.0002451	0.95	1.33490000000000
1.50E-04	2.00E+12	1.70E-04	1.36	0.000260419	0.95	1.33780000000000
1.50E-04	2.00E+12	1.80E-04	1.36	0.000275738	0.95	1.34030000000000
1.50E-04	2.00E+12	1.90E-04	1.36	0.000291056	0.95	1.34250000000000
1.50E-04	2.00E+12	2.00E-04	1.36	0.000306375	0.95	1.34440000000000
1.50E-04	2.00E+12	2.10E-04	1.36	0.000321694	0.95	1.34600000000000
1.50E-04	2.00E+12	1.20E-04	1.38	0.000190598	0.985	1.33510000000000
1.50E-04	2.00E+12	1.30E-04	1.38	0.000206481	0.985	1.34120000000000
1.50E-04	2.00E+12	1.40E-04	1.38	0.000222364	0.985	1.34620000000000
1.50E-04	2.00E+12	1.50E-04	1.38	0.000238247	0.985	1.35030000000000
1.50E-04	2.00E+12	1.60E-04	1.38	0.00025413	0.985	1.35380000000000
1.50E-04	2.00E+12	1.70E-04	1.38	0.000270013	0.985	1.35680000000000
1.50E-04	2.00E+12	1.80E-04	1.38	0.000285896	0.985	1.35930000000000
1.50E-04	2.00E+12	1.90E-04	1.38	0.000301779	0.985	1.36150000000000
1.50E-04	2.00E+12	2.00E-04	1.38	0.000317663	0.985	1.36340000000000

1.50E-04	2.00E+12	2.10E-04	1.38	0.000333546	0.985	1.36500000000000
1.50E-04	2.00E+12	1.20E-04	1.4	0.000190598	0.985	1.35390000000000
1.50E-04	2.00E+12	1.30E-04	1.4	0.000206481	0.985	1.36010000000000
1.50E-04	2.00E+12	1.40E-04	1.4	0.000222364	0.985	1.36520000000000
1.50E-04	2.00E+12	1.50E-04	1.4	0.000238247	0.985	1.36940000000000
1.50E-04	2.00E+12	1.60E-04	1.4	0.00025413	0.985	1.37290000000000
1.50E-04	2.00E+12	1.70E-04	1.4	0.000270013	0.985	1.37590000000000
1.50E-04	2.00E+12	1.80E-04	1.4	0.000285896	0.985	1.37850000000000
1.50E-04	2.00E+12	1.90E-04	1.4	0.000301779	0.985	1.38070000000000
1.50E-04	2.00E+12	2.00E-04	1.4	0.000317663	0.985	1.38260000000000
1.50E-04	2.00E+12	2.10E-04	1.4	0.000333546	0.985	1.38430000000000

imag-x pol	area-x pol	power-x pol	sens-x pol
			#DIV/0!
2.8911000000000E-07	4.8536000000000E-08	8.8997000000000E-01	0.9182065782329
1.4685000000000E-07	5.4519000000000E-08	8.9947000000000E-01	0.9239226907631
3.0408000000000E-07	6.0673000000000E-08	9.0636000000000E-01	0.9275614035088
4.3320000000000E-07	6.6891000000000E-08	9.1131000000000E-01	0.9297654955508
5.2816000000000E-07	7.3018000000000E-08	9.1468000000000E-01	0.9308473486877
2.0142000000000E-06	7.9385000000000E-08	9.1650000000000E-01	0.9306344480073
5.6690000000000E-07	8.4697000000000E-08	9.1782000000000E-01	0.9301993446620
1.0320000000000E-06	9.0100000000000E-08	9.1758000000000E-01	0.9283996957018
4.0952000000000E-07	9.5026000000000E-08	9.1621000000000E-01	0.9256755545427
5.2101000000000E-07	9.8439000000000E-08	9.1446000000000E-01	0.9227158789166
			#DIV/0!
			#DIV/0!
			#DIV/0!
1.6689000000000E-07	4.7887000000000E-08	9.0380000000000E-01	0.9333205844106
3.6122000000000E-08	5.4087000000000E-08	9.1270000000000E-01	0.9382767286019
1.8101000000000E-08	6.0508000000000E-08	9.1942000000000E-01	0.9417427921093
3.3738000000000E-08	6.7158000000000E-08	9.2428000000000E-01	0.9437848876787
5.2403000000000E-08	7.3883000000000E-08	9.2786000000000E-01	0.9450101848359
4.2523000000000E-08	8.0562000000000E-08	9.3043000000000E-01	0.9455589430894
3.2902000000000E-07	8.7233000000000E-08	9.3185000000000E-01	0.9452231572620
1.5043000000000E-07	9.3534000000000E-08	9.3278000000000E-01	0.9446759189797
2.8393000000000E-07	9.9601000000000E-08	9.3271000000000E-01	0.9431898876405
1.1238000000000E-07	1.0500000000000E-07	9.3211000000000E-01	0.9414548107138
			#DIV/0!
			#DIV/0!
4.0478000000000E-08	4.7553000000000E-08	9.0979000000000E-01	0.9398514242309
3.1116000000000E-08	5.3820000000000E-08	9.1840000000000E-01	0.9445130066546
7.4549000000000E-09	6.0373000000000E-08	9.2489000000000E-01	0.9476046406509
6.6304000000000E-09	6.7160000000000E-08	9.2979000000000E-01	0.9497629562866
1.6981000000000E-08	7.4108000000000E-08	9.3340000000000E-01	0.9509506330062
1.2652000000000E-08	8.1100000000000E-08	9.3610000000000E-01	0.9516340260129
1.7443000000000E-08	8.8095000000000E-08	9.3787000000000E-01	0.9516550026114
1.9119000000000E-07	9.4995000000000E-08	9.3887000000000E-01	0.9511085288641
6.4667000000000E-08	1.0157000000000E-07	9.3934000000000E-01	0.9502398095805
1.4487000000000E-07	1.0779000000000E-07	9.3913000000000E-01	0.9488980683507
1.6311000000000E-09	4.6865000000000E-08	9.2026000000000E-01	0.9512087484084
2.0566000000000E-09	5.3205000000000E-08	9.2839000000000E-01	0.9552476886370
4.5797000000000E-09	5.9929000000000E-08	9.3452000000000E-01	0.9579836577032
9.1173000000000E-10	6.6956000000000E-08	9.3924000000000E-01	0.9598986891802
5.4073000000000E-10	7.4252000000000E-08	9.4287000000000E-01	0.9611172994534
1.0498000000000E-09	8.1742000000000E-08	9.4562000000000E-01	0.9617892099057
1.4728000000000E-09	8.9351000000000E-08	9.4772000000000E-01	0.9621522842640
9.1732000000000E-10	9.7033000000000E-08	9.4915000000000E-01	0.9620470069776
2.3495000000000E-09	1.0467000000000E-07	9.5009000000000E-01	0.9616577673463

2.0180000000000E-08	1.1220000000000E-07	9.5054000000000E-01	0.9609854945055
1.3994000000000E-10	4.6178000000000E-08	9.2910000000000E-01	0.9607356525593
6.7317000000000E-11	5.2560000000000E-08	9.3674000000000E-01	0.9642202779207
6.0561000000000E-11	5.9373000000000E-08	9.4253000000000E-01	0.9665558159977
1.5438000000000E-10	6.6547000000000E-08	9.4705000000000E-01	0.9682123557763
1.6343000000000E-10	7.4064000000000E-08	9.5060000000000E-01	0.9693641197465
4.3472000000000E-11	8.1874000000000E-08	9.5336000000000E-01	0.9700588705575
4.2041000000000E-11	8.9926000000000E-08	9.5556000000000E-01	0.9704635473341
1.0609000000000E-10	9.8202000000000E-08	9.5720000000000E-01	0.9705801405084
7.7612000000000E-11	1.0660000000000E-07	9.5843000000000E-01	0.9704918269926
5.8935000000000E-11	1.1510000000000E-07	9.5929000000000E-01	0.9701697608900

EML-x pol	confinement -x pol	NA- x pol	spot-x pol
	#DIV/0!	#DIV/0!	#DIV/0!
0.0103690000000	1.051882053510E-01	3.58588359391236E-01	2.099936554039E-04
0.0100350000000	5.342910295664E-02	3.40753454925541E-01	2.257193648236E-04
0.0098603000000	1.106348084920E-01	3.24929425054238E-01	2.425499226572E-04
0.0098116000000	1.576131249630E-01	3.10988705277793E-01	2.601353905925E-04
0.0098383000000	1.921628533713E-01	2.98870402850140E-01	2.780456874782E-04
0.0100610000000	7.328355408598E-01	2.87666915648951E-01	2.976762898033E-04
0.0102620000000	2.062578036508E-01	2.79225490834259E-01	3.186783847607E-04
0.0106470000000	3.754772506044E-01	2.71359053639503E-01	3.417655980809E-04
0.0111760000000	1.489975229336E-01	2.64737802385841E-01	3.666081387283E-04
0.0117610000000	1.895614363734E-01	2.60424530950661E-01	3.947329988659E-04
	#DIV/0!	#DIV/0!	#DIV/0!
	#DIV/0!	#DIV/0!	#DIV/0!
	#DIV/0!	#DIV/0!	#DIV/0!
0.0090694000000	6.072034724163E-02	3.60695955542145E-01	2.025430842259E-04
0.0087157000000	1.314243144024E-02	3.41953041395841E-01	2.176244016960E-04
0.0084923000000	6.585769101928E-03	3.25325322413265E-01	2.329337148806E-04
0.0083769000000	1.227504988458E-02	3.10429577555983E-01	2.492148856553E-04
0.0083320000000	1.906602166998E-02	2.97271189107287E-01	2.657669908622E-04
0.0083631000000	1.547133636381E-02	2.85730572587856E-01	2.829893498560E-04
0.0085159000000	1.197088420483E-01	2.75449140432706E-01	3.010262731485E-04
0.0086764000000	5.473163062831E-02	2.66691884916084E-01	3.191982506561E-04
0.0089652000000	1.033035424071E-01	2.59003433062438E-01	3.405100089116E-04
0.0093174000000	4.088772618500E-02	2.52692893462028E-01	3.608054777073E-04
	#DIV/0!	#DIV/0!	#DIV/0!
	#DIV/0!	#DIV/0!	#DIV/0!
0.0085062000000	1.472729471896E-02	3.61795190246181E-01	1.994319052736E-04
0.0081684000000	1.132107570718E-02	3.42700817864242E-01	2.139614841489E-04
0.0079286000000	2.712350150708E-03	3.25650317017115E-01	2.294335509974E-04
0.0077636000000	2.412368568224E-03	3.10425400705911E-01	2.446111211183E-04
0.0077071000000	6.178274411350E-03	2.96859398701416E-01	2.609428181857E-04
0.0076794000000	4.603234665355E-03	2.84858407350249E-01	2.773234860579E-04
0.0077545000000	6.346365971213E-03	2.74200009825249E-01	2.943521707151E-04
0.0078956000000	6.956152668900E-02	2.64777967293407E-01	3.124140567000E-04
0.0080836000000	2.352808853181E-02	2.56647604614045E-01	3.311060130401E-04
0.0083387000000	5.270871055722E-02	2.49607501270249E-01	3.497343766808E-04
0.0075182000000	5.934505266093E-04	3.64091522220405E-01	1.941879853992E-04
0.0071552000000	7.482621255746E-04	3.44442059620927E-01	2.084524562214E-04
0.0069070000000	1.666253066466E-03	3.26726097722395E-01	2.229894381123E-04
0.0067260000000	3.317188698581E-04	3.10852309855941E-01	2.375897468232E-04
0.0066032000000	1.967362536040E-04	2.96596749182764E-01	2.527452518057E-04
0.0065425000000	3.819535055082E-04	2.83828023220089E-01	2.684342918528E-04
0.0065164000000	5.358555181106E-04	2.72409975353479E-01	2.840381534384E-04
0.0065592000000	3.337527049655E-04	2.62175719039033E-01	3.003754049307E-04
0.0066403000000	8.548292638517E-04	2.53065448998348E-01	3.170617566578E-04

0.0067679000000	7.342181121314E-03	2.44953050330988E-01	3.334956737221E-04
0.0066874000000	5.091500624959E-05	3.66428706590057E-01	1.898664794393E-04
0.0063154000000	2.449225007649E-05	3.46297059970986E-01	2.037134056996E-04
0.0060628000000	2.203418388939E-05	3.28088402511102E-01	2.178390699693E-04
0.0058610000000	5.616877708169E-05	3.11713553329656E-01	2.320688217690E-04
0.0057090000000	5.946147971538E-05	2.96939792000692E-01	2.464001758981E-04
0.0056097000000	1.581661534716E-05	2.83617550972699E-01	2.611234749103E-04
0.0055313000000	1.529596811304E-05	2.71602108191002E-01	2.763278536541E-04
0.0055109000000	3.859920689595E-05	2.60717258879560E-01	2.915325832840E-04
0.0055167000000	2.823792671890E-05	2.50909607002067E-01	3.069340906536E-04
0.0055461000000	2.144258891896E-05	2.42030526587941E-01	3.230494682651E-04
